# Supplementary material for: Practical selection of representative sets of RNA-seq samples using a hierarchical approach
Source: Bioinformatics. 2021 Jul 12;37(Suppl 1):i334–41. doi: 10.1093/bioinformatics/btab315 (PMC8275344; doi:10.1093/bioinformatics/btab315)
Supplement: btab315_Supplementary_Data [file btab315_supplementary_data.pdf]

Supplementary Materials

Practical selection of representative sets of RNA-seq samples using a hierarchical approach

Laura H. Tung[1] and Carl Kingsford[1*]

[1]Computational Biology Department, School of Computer Science, Carnegie Mellon University, Pittsburgh, PA 15213, USA

[*]To whom correspondence should be addressed: carlk@cs.cmu.edu

1 Supplementary tables and figures

Table S1 *– Partial Hausdorff distance, classical Hausdorff distance, runtime, and memory usage of direct apricot, hierarchical selection, and random selection, using the most recent 1000, 2000, 5000, 8000, 10000, 12000 samples in the SRA as the full sets.*

Set of samples	Method	Hausdorff d_H	Partial Hausdorff d_{HK}	Runtime (seconds) Real	User	Sys	Memory (GB)
Recent 1000	direct apricot	0.688321022	0.685257115	174.58	11070.16	111.42	17.131
(select 100,	hierarchical (seeded)	0.578381536	0.569809642	118.91	3449.28	292.08	up to 8.061
$l = 5$)	random selection	0.837951394	0.826812255	0.424	2.282	5.205	negligible
Recent 2000	direct apricot	0.664517495	0.662815581	655.18	42882.64	286.79	29.223
(select 200,	hierarchical (seeded)	0.650712434	0.637185175	240.77	6906.76	586.80	up to 10.077
$l = 10$)	random selection	0.894947772	0.848467165	0.421	2.157	5.323	negligible
Recent 5000	direct apricot	0.624796548	0.62353618	4334.92	306588.88	1323.23	67.515
(select 500,	hierarchical (seeded)	0.696114667	0.675768259	717.42	35803.41	1164.84	up to 15.115
$l = 10$)	random selection	0.973292492	0.896965855	0.423	2.241	5.242	negligible
Recent 8000	direct apricot	0.610919893	0.610735987	10095.85	701276.54	1513.85	101.776
(select 800,	hierarchical (seeded)	0.760030601	0.679049314	1545.26	84481.00	1588.09	up to 22.169
$l = 10$)	random selection	0.991018754	0.904692245	0.536	2.347	5.129	negligible
Recent 10000	direct apricot	0.607682609	0.607369442	14768.33	1047257.38	2273.86	118.906
(select 1000,	hierarchical (seeded)	0.70951932	0.665272021	2103.18	123182.85	1749.27	up to 26.200
$l = 10$)	random selection	0.994547626	0.986377775	0.439	2.420	5.079	negligible
Recent 12000	direct apricot	0.598587653	0.598052637	20902.48	1449102.48	2202.62	142.083
(select 1200,	hierarchical (seeded)	0.770540655	0.652204188	2914.35	175006.16	2103.21	up to 27.207
$l = 10$)	random selection	0.994469360	0.985777326	0.49	2.57	4.97	negligible

Table S2 *– Partial Hausdorff distance, classical Hausdorff distance, runtime, and memory usage of direct apricot, hierarchical selection, and random selection, using the early-time 1000, 2000, 5000, 8000, 10000, 12000 samples in the SRA as the full sets.*

**Set of samples**	**Method**	**Hausdorff d_H**	**Partial Hausdorff d_{HK}**	**Runtime (seconds) Real**	**User**	**Sys**	**Memory (GB)**
Early 1000	direct apricot	0.725003411	0.723976221	153.22	7701.56	125.76	11.084
(select 100,	hierarchical (seeded)	0.745518016	0.725044029	100.12	2351.73	215.52	up to 5.038
$l = 5$)	random selection	0.950139546	0.938242439	0.43	2.27	5.21	negligible
Early 2000	direct apricot	0.67307199	0.672427473	401.49	22979.13	218.19	17.131
(select 200,	hierarchical (seeded)	0.760020571	0.706453874	187.64	4360.50	383.16	up to 6.046
$l = 10$)	random selection	0.872261407	0.838447692	0.65	2.43	5.07	negligible
Early 5000	direct apricot	0.61634404	0.615099659	2298.45	133723.63	565.12	39.300
(select 500,	hierarchical (seeded)	0.666160711	0.659981919	497.60	21561.05	1262.98	up to 9.069
$l = 10$)	random selection	0.938632619	0.935902009	0.45	2.37	5.14	negligible
Early 8000	direct apricot	0.630098995	0.628989605	5719.26	382221.70	874.59	70.538
(select 800,	hierarchical (seeded)	0.834517734	0.71208656	1145.94	60263.98	1509.99	up to 16.123
$l = 10$)	random selection	0.9916713	0.908157434	0.78	1.55	3.35	negligible
Early 10000	direct apricot	0.639209628	0.6383828	12315.44	800933.84	1446.46	89.684
(select 1000,	hierarchical (seeded)	0.837916798	0.713645489	1648.26	95117.21	1381.86	up to 19.146
$l = 10$)	random selection	0.99772421	0.990244824	1.38	2.18	2.97	negligible
Early 12000	direct apricot	0.635004017	0.634814991	14554.59	984029.09	1247.92	110.845
(select 1200,	hierarchical (seeded)	0.908677303	0.709577599	2290.33	137885.59	1681.81	up to 22.169
$l = 10$)	random selection	0.999260054	0.989801358	0.53	2.34	5.25	negligible

Table S3 *– Partial Hausdorff distance, classical Hausdorff distance, runtime, and memory usage of direct apricot, hierarchical selection, and random selection, using the mid-time 1000, 2000, 5000, 8000, 10000, 12000 samples in the SRA as the full sets.*

**Set of samples**	**Method**	**Hausdorff d_H**	**Partial Hausdorff d_{HK}**	**Runtime (seconds) Real**	**User**	**Sys**	**Memory (GB)**
Mid 1000	direct apricot	0.666185918	0.664052819	150.21	9130.84	126.60	14.108
(select 100,	hierarchical (seeded)	0.685598613	0.648069325	160.45	2705.68	252.78	up to 6.046
$l = 5$)	random selection	0.968587397	0.753610206	0.43	2.45	5.03	negligible
Mid 2000	direct apricot	0.651423605	0.648054888	545.46	34587.00	248.80	25.192
(select 200,	hierarchical (seeded)	0.732927883	0.676261249	243.70	5827.03	508.06	up to 9.069
$l = 10$)	random selection	0.967519071	0.923284417	0.43	2.15	5.24	negligible
Mid 5000	direct apricot	0.646540816	0.646398158	3084.32	209037.81	892.53	55.422
(select 500,	hierarchical (seeded)	0.719052575	0.676710291	626.75	28509.77	983.47	up to 12.092
$l = 10$)	random selection	0.961274547	0.867699133	0.45	2.34	5.17	negligible
Mid 8000	direct apricot	0.636313583	0.635731787	7142.40	464515.34	865.10	81.622
(select 800,	hierarchical (seeded)	0.943464491	0.686230795	1262.73	64248.73	1301.38	up to 18.138
$l = 10$)	random selection	0.998728362	0.989508253	2.43	3.14	2.91	negligible
Mid 10000	direct apricot	0.654537313	0.653742747	12582.81	780770.97	1574.01	100.768
(select 1000,	hierarchical (seeded)	0.807148615	0.734005	1818.98	98855.02	1548.71	up to 22.169
$l = 10$)	random selection	0.999886845	0.997812734	0.45	2.42	4.99	negligible
Mid 12000	direct apricot	0.642698074	0.642257142	16318.41	1063199.56	1357.40	123.945
(select 1200,	hierarchical (seeded)	0.758132270	0.662355963	2529.71	146293.35	1731.62	up to 23.177
$l = 10$)	random selection	0.999041296	0.995426952	0.49	2.35	5.20	negligible

Table S4 *– Selecting different sizes of representative sets from the SRA entire set (N=196523 human RNA-seq samples): partial Hausdorff distance and classical Hausdorff distance of hierarchical selection and random selection.*

**Metric**	**Select 3000**		**Select 4000**		**Select 5000**		**Select 7000**	
	hierarchical (seeded)	random selection	hierarchical (seeded)	random selection	hierarchical (seeded)	random selection	hierarchical (seeded)	random selection
Hausdorff d_H	0.945978504	0.998763361	0.945978504	0.998763361	0.945978504	0.998763361	0.875009817	0.998763361
Partial Hausdorff d_{HK}	0.844519904	0.997721045	0.844274154	0.997718649	0.844274154	0.997718649	0.753391502	0.997718289
Representative -set-size/ Full-set-size	0.0153		0.0204		0.0254		0.0356	

Table S5 *– Performance comparison of hierarchical selection using seeded-chunking method vs. using sequential chunking method: partial Hausdorff distance and classical Hausdorff distance, using the most recent 1000, 2000, 5000, 8000, 10000 samples in the SRA as the full sets.*

Set of samples	Method	Hausdorff d_H	Partial Hausdorff d_{HK}
Recent 1000	hierarchical (seeded)	0.578381536	0.569809642
(select 100, $l = 5$)	hierarchical (sequential)	0.612622717	0.578381536
Recent 2000	hierarchical (seeded)	0.650712434	0.637185175
(select 200, $l = 10$)	hierarchical (sequential)	0.656343899	0.643755957
Recent 5000	hierarchical (seeded)	0.696114667	0.675768259
(select 500, $l = 10$)	hierarchical (sequential)	0.72711968	0.708127649
Recent 8000	hierarchical (seeded)	0.760030601	0.679049314
(select 800, $l = 10$)	hierarchical (sequential)	0.770009257	0.754661415
Recent 10000	hierarchical (seeded)	0.70951932	0.665272021
(select 1000, $l = 10$)	hierarchical (sequential)	0.790246213	0.752879052

In Tables S1, S2, and S3, for N=1000, 2000, 5000, d_{HK} is the 3rd largest distance; for N=8000, 10000, d_{HK} is the 4th largest distance; for N=12000, d_{HK} is the 5th largest distance. The results are from single runs.

The SRA accession list of the full set obtained using the Entrez API is in the reverse order of accession numbers.

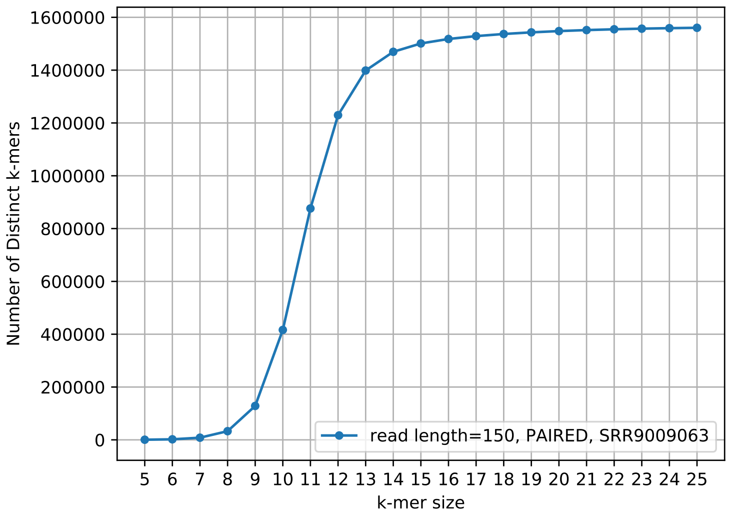

Figure S1 *– The number of distinct k-mers vs. k-mer size: read-length=150, paired-end reads, from SRR9009063 (10000 random reads).*

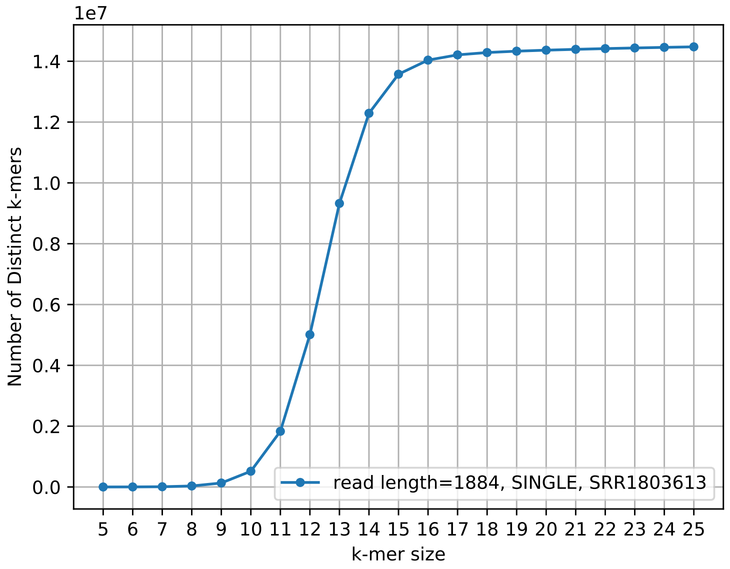

Figure S2 *– The number of distinct k-mers vs. k-mer size: read-length=1884, single-end reads, from SRR1803613 (10000 random reads).*

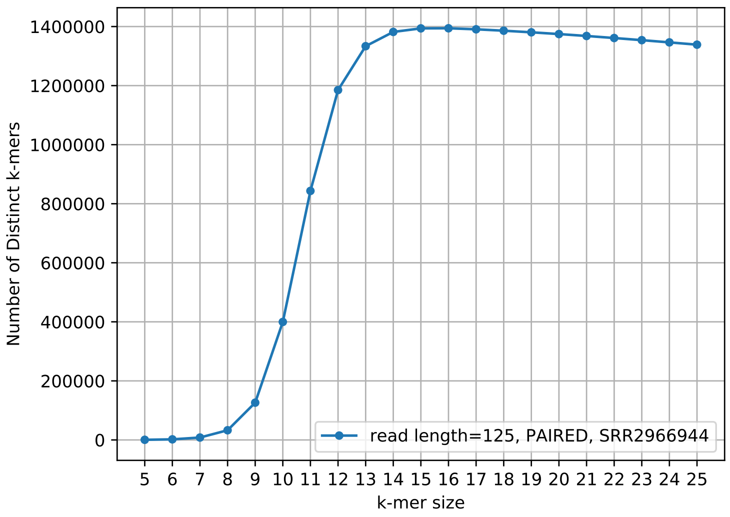

Figure S3 *– The number of distinct k-mers vs. k-mer size: read-length=125, paired-end reads, from SRR2966944 (10000 random reads).*

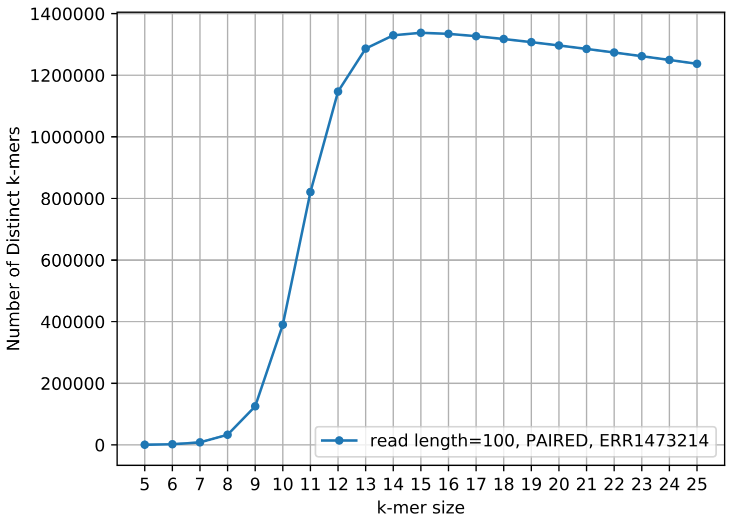

Figure S4 *– The number of distinct k-mers vs. k-mer size: read-length=100, paired-end reads, from ERR1473214 (10000 random reads).*

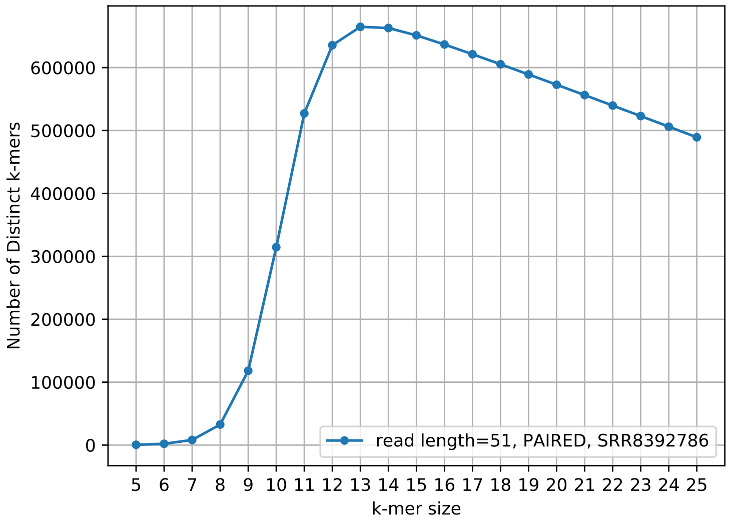

Figure S5 *– The number of distinct k-mers vs. k-mer size: read-length=51, paired-end reads, from SRR8392786 (10000 random reads).*

In Figs. S3, S4, and S5, the horizontal part of the curve bends down as k-mer size further increases, especially for shorter read-lengths, since when read-lengths are short, using larger k-mers would reduce the number of distinct k-mers.

Table S6 *– Hardware specifications of the system on which the experiments were run.*

Attribute	Value
Architecture	x86_64
CPU op-mode(s)	32-bit, 64-bit
Byte Order	Little Endian
CPU(s)	88
On-line CPU(s) list	0-87
Thread(s) per core	2
Core(s) per socket	22
Socket(s)	2
NUMA node(s)	2
Vendor ID	GenuineIntel
CPU family	6
Model	79
Model name	Intel(R) Xeon(R) CPU E5-2699A v4 @ 2.40GHz
Stepping	1
CPU MHz	2871.660
CPU max MHz	3600.0000
CPU min MHz	1200.0000
BogoMIPS	4793.95
Virtualization	VT-x
Mem Total	1056631884 kB

Table S7 *– Performance comparison of using different chunk size m in hierarchical selection, using the most recent 1000 samples in the SRA as the full set. Partial Hausdorff distance and classical Hausdorff distance.*

Metric	hierarchical (seeded) $m = 100$	hierarchical (seeded) $m = 150$	hierarchical (seeded) $m = 200$	direct apricot
Hausdorff d_H	0.744303019	0.687825966	0.578381536	0.688321022
Partial Hausdorff d_{HK}	0.725728616	0.684475004	0.569809642	0.685257115

Table S7 shows the impact of the chunk size m on the selection accuracy of hierarchical selection, illustrating that chunk size m needs to be large enough to avoid chunk overlaps in the seeded-chunking. When $m = 100$, hierarchical selection performs worse than direct apricot. When $m = 150$, hierarchical selection performs almost as equally as (slightly better than) direct apricot. When $m = 200$, hierarchical selection performs better than direct apricot. Smaller chunks have more overlaps which decrease the selection accuracy.

2 Additional discussions

We compared the performance between direct apricot and hierarchical selection by using the most recent, early-time, and mid-time RNA-seq samples in the SRA as the full sets. A more robust comparison would be computing confidence intervals for partial Hausdorff distances for each N value and compare the confidence intervals between different methods. Computing confidence intervals would involve running direct apricot and hierarchical selection on a substantial number of randomly selected subsets (used as the "full sets") across the SRA spectrum for each N value. Each randomly selected subset starts from a randomly selected position on the SRA accession list and contains N consecutive samples starting from that position; the subset should not contain randomly selected N samples, since we use the subset as the full set (we found that randomly

selected N samples have a much smaller number of samples that are similar to each other and thus are not suitable to be used as a "full set"). This is a direction for future analysis.

We download 10,000 reads from each RNA-seq sample to represent that sample. We chose this number since prior researchers found that 10,000 reads are sufficient to tell what genome this sample belongs to, and we do not have extra disk space to hold more reads from all RNA-seq samples in the SRA. We envision that downloading more reads (such as 100,000 reads) would represent each sample better, and thus the selected representative samples based on the subsets of reads would better represent the SRA full set. In order to evaluate the effect of downloading different numbers of reads, one would need to download all reads (rather than the subset of reads) from each of the RNA-seq samples in the full set. Currently, the partial Hausdorff distance is computed based on the k-mer similarities using the subset of reads; this is effective for assessing the representative set selection algorithm itself. However, to assess the effect of downloading different numbers of reads, the partial Hausdorff distance would need to be computed using all reads from each of the RNA-seq samples in the full set. In this assessment, although the representative set is selected based on the subset of reads (10K or 100K reads), in order to compare which representative set represents the RNA-seq full set better, one would need to use all reads from each sample to compute the partial Hausdorff distance. The comparison between the d_{HK} value computed using 10K reads and the d_{HK} value computed using 100K reads would not be meaningful since they do not share the common ground. Thus, in this specific case, only the comparison between the d_{HK} values computed using all reads of each sample can tell which representative set represents the RNA-seq full set better. Let us show this proposed evaluation process in more detail. First, one would select the representative set using 10K reads from each sample; one would then compute the d_{HK} based on the k-mer similarities using all reads from each of the selected representative samples and all reads from each sample in the full set. Second, one would select the representative set using 100K reads from each sample; one would then compute the d_{HK} based on the k-mer similarities using all reads from each of the selected representative samples and all reads from each sample in the full set. Then, the d_{HK} values of these two cases can be compared to assess the effect of downloading 10K reads vs. downloading 100K reads. This is a direction for future analysis when extra disk space becomes available for downloading all reads from each of the RNA-seq samples in the full set.

Bioinformatics tools should be validated and optimized on varying cell/tissue types and experiments. Thus, our method takes in all available RNA-seq samples regardless of their tissue/cell types, treatments, or experiments to select a representative set. However, our method can also be adapted for selecting representative samples for specific tissues/cell types. This only needs to be done at the full set construction stage. Our current SRA full set contains all RNA-seq samples (excluding those non-public, aligned, or with no valid 17-mers). From the full SRA accession list, one can extract those RNA-seq samples of specific tissues/cell types based on their BioSample Attributes. These extracted samples of specific tissues/cell types can be used as the full set, and then our hierarchical representative set selection can be performed to get representative samples for specific tissues/cell types.

When we download the subset of reads for each RNA-seq sample, we skip the first 5000 reads, since the beginning of sequencing may contain some technical variation of signal introduced in the sequencing process, as suggested by prior researchers. We also filter out the technical reads, remove those tags' sequences, and filter out reads that are all N's. When we do k-mer counting using Jellyfish, k-mers with N's in the middle are skipped; however, bases with low quality scores

are still counted into k-mers, as Jellyfish does not read or consider the quality scores. However, using k-mers instead of the original sequences to compute the similarity between samples is more resilient to sequencing errors. Our choice of optimal k-mer size 17 is to ensure that the k-mer matches move from random to a representative of reads' content and are still resilient to sequencing errors. One potential improvement that could be made in Jellyfish would be taking into account the base call quality scores for each read to filter out those k-mers containing many low-quality bases (below a quality score threshold). We may also filter out or trim those reads with many low-quality bases (below a quality score threshold) before doing k-mer counting. These are directions for future work.

The hierarchical selection is more accurate than direct apricot for the most recent 1000 and 2000 samples and the mid-time 1000 samples. For the early-time 1000 samples, the hierarchical selection is nearly as accurate as direct apricot. It seems that for smaller N's, the hierarchical selection may have some advantage. A possible reason for the hierarchical selection being more accurate than direct apricot in these cases is that the chunks generated by the seeded-chunking method may have minimal or no overlaps. When chunks have no overlaps, the union of the representative sets selected from every chunk would be similar to the representative set selected directly from the original full set. Given that chunks have no overlaps, since the hierarchical selection performs the 2nd-round of representative set selection – selecting representative samples from the merged set (in the one-level hierarchy), it could be possible that the final representative set is better than the representative set selected directly from the original full set, since the direct selection only performs one round of selection. This is also supported by Table S7: for the same "Recent 1000" samples, when chunks are large enough to avoid overlaps ($m = 200$; $m = 150$), the hierarchical selection is more accurate than direct apricot; when the chunk size is small ($m = 100$) such that there are chunk overlaps, the hierarchical selection is less accurate than direct apricot. The selection accuracy loss of the hierarchical selection mainly comes from chunk overlaps. In our observation, smaller full sets (e.g. $N = 1000$) are more likely to have some clustered structures than large full sets, and thus would be more likely to generate chunks with no overlaps (when the chunk size is large enough).

When we get the SRA full set, we exclude those non-public samples as we do not have access to them; we are testing on what is publicly available. It is also more convenient for tools' developers to use publicly available representative samples to evaluate bioinformatics tools. It is possible that there might be a difference in sample distribution if those non-public samples were in the full set. By the same reasoning, some non-public samples might not be sufficiently represented by the representative set selected from publicly available samples only.
